# Martial Arts and Metabolic Diseases

**DOI:** 10.3390/sports4020028

**Published:** 2016-05-09

**Authors:** Hidetaka Hamasaki

**Affiliations:** 1 Department of Internal Medicine, National Center for Global Health and Medicine Kohnodai Hospital, 1-7-1 Kohnodai, Chiba 272-8516, Japan; hhamasaki78@gmail.com; Tel.: +81-47-372-3501; Fax: +81-47-372-1858; 2 Hamasaki Clinic, 2-21-4 Nishida, Kagoshima 890-0046, Japan

**Keywords:** martial arts, tai chi, metabolic disease, diabetes

## Abstract

Different forms of martial arts are practiced worldwide, each with various intensities of physical activity. These disciplines are potentially an effective exercise therapy for metabolic diseases. Tai chi is the most well-studied style of martial arts and has shown evidence of its effect on metabolic diseases; however, little evidence is available regarding the association between other styles of martial arts and metabolic health. To summarize and evaluate the effects of martial arts on metabolic diseases, eligible articles were searched by using Pubmed. To date, systematic reviews provide no definite conclusion on the effectiveness of tai chi for treating metabolic diseases because of a small numbers of subjects, short durations of clinical trials, and some biases involved in testing. However, there are several clinical studies on subjects with metabolic diseases, which show that tai chi improves obesity, glycemic control, blood pressure control, and lipid profiles. Currently, some limited evidence suggests that other martial arts, such as kung fu and karate, may be beneficial for body composition, glycemic control, and arterial stiffness. To clarify the effectiveness of martial arts for treating metabolic diseases, well-designed prospective studies, preferably with a larger number of subjects and of longer duration, are warranted.

## 1. Introduction

Many studies evaluating the benefits of physical activity on health generally recommend 30–60 min/day of moderate to vigorous intensity of physical activity for at least five days/week [1]. Such physical activity intensity is classified using metabolic equivalents (METs), *i.e.*, 3.0–5.9 METs, including various sports activities (Table 1) [1,2]. Conversely, physical inactivity is one of the leading causes of mortality, and in fact, 31% of people cannot achieve the recommended level of physical activity [3].

Judo, karate, aikido, kung fu, and tai chi are examples of some of the various styles of martial arts. In addition, the intensity of physical activity involved in martial arts varies according to its style. Performing tai chi is equal to 1.5–3.0 METs (light to moderate intensity), practicing various martial arts at a slow pace is equal to 5.3 METs (moderate intensity), and practicing combat martial arts, such as judo, karate, and kick boxing, at a moderate pace is equal to 10.3 METs (vigorous intensity) [2]. In terms of physical activity for health, martial arts have proven to provide beneficial effects on the human body. It has been reported that tai chi has beneficial effects on blood pressure, muscle strength, and the subject’s ability to maintain balance [4]. Recent studies have shown that tai chi also improves glycemic control [5], health-related quality of life (HR-QOL) [6], and peripheral nerve conduction velocity [7] as well as reducing oxidative stress [8] and improving immunity in patients with type 2 diabetes [9]. Tai chi is the most well-studied style of martial arts, and there is sufficient evidence for its effect on metabolic diseases; however, little evidence is available regarding the association between other styles of martial arts and metabolic health.

This review is aimed at summarizing and evaluating the effects of tai chi and other martial arts on metabolic diseases, providing clinicians and patients with new insights into the strategy of using martial arts as an exercise therapy for metabolic diseases.

## 2. Methods

I searched literature written in English on martial arts and metabolic diseases by using Pubmed from its inception to March 2016. I used the following combined Medical Subject Headings (MeSH) terms. Search terms were “martial arts”, “tai ji [MeSH] or tai chi“, “metabolic disease”, “diabetes mellitus”, “hypertension”, and “dyslipidemias”. I aimed primarily at reviewing controlled trials, but also included reviews and other types of clinical studies such as before-after studies, case-control studies, and cross-sectional studies. I found 61 published articles, and the titles and abstracts of identified articles were reviewed to determine their relevance. Twenty-seven articles were eligible. Clinical studies which did not have study participants with metabolic diseases or outcomes related to metabolic diseases were excluded.

## 3. Tai Chi

Tai chi is an ancient Chinese martial art, which is characterized by slow, smooth, and fluid movements. The level of physical activity intensity of tai chi depends on its style, form, and duration. The most popular is the classical Yang style, which is a moderate intensity exercise [10]. The efficacy and safety of tai chi have been investigated in several clinical studies, as well as in the publication of several systematic reviews. Hartley *et al.* evaluated the effectiveness of tai chi in the prevention of cardiovascular disease (CVD) by researching previous randomized controlled trials (RCTs) of tai chi in healthy subjects and individuals at a high risk for CVD, without language restrictions. Although some trials showed that tai chi lowered blood pressure and ameliorated lipid profiles, none showed consistent beneficial effects of tai chi on CVD risk factors. The authors pointed out some of the potential problems with the study, including small numbers of subjects, short durations of the trials, and some risk of bias [11]. On the other hand, another systematic review to investigate the effectiveness of tai chi for the primary prevention of stroke in middle-aged and elderly individuals revealed that tai chi intervention significantly reduced the incidence of nonfatal stroke (relative risk = 0.11, 95% CI; 0.01–0.85) and cardio-cerebrovascular disease (relative risk = 0.33, 95% CI; 0.11–0.96). This study also demonstrated that tai chi exercise was associated with the improvement of metabolic diseases such as obesity, hypertension, dyslipidemia, and diabetes [12]. Lee *et al.* [13] independently evaluated the systematic reviews of tai chi because the previous RCTs had different characteristics of the study population, and their conclusions were contradictory. Although the evidence for fall prevention, improving psychological health, and health benefits for the elderly was relatively clear, the evidence for improving metabolic diseases was not definitive [13]. Recently, Yang *et al.* [14] evaluated a total of 507 clinical studies, including systematic reviews, RCTs, observational studies, case series, and case reports, to summarize the evidence of the effect of tai chi on health. Among the 507 studies, 317 were conducted in China (62.5%) with various tai chi styles for interventions (Yang style, 72.84%; Chen style, 3.88%; Sun style, 4.53%; Wu style, 1.9%; and others, 23.71%), and the duration of the tai chi interventions varied from five days to three years. These wide variations in style, intensity, duration, and practicing methods of the interventions may demonstrate the limitations of the previous studies. They concluded that further well-designed studies would be required to confirm the effects of tai chi for diseases.

A few systematic reviews evaluated the effectiveness of tai chi for type 2 diabetes. Lee *et al.* investigated whether tai chi reduced fasting blood glucose levels and glycosylated hemoglobin A1c (HbA1c) in RCTs and controlled clinical trials. The meta-analysis failed to show beneficial effects of tai chi on glycemic control; therefore, it may be concluded that tai chi is not an effective therapy for type 2 diabetes [15,16]. In systematic reviews, evidence that tai chi is effective for improving metabolic diseases, such as hypertension, dyslipidemia, and type 2 diabetes, is controversial. However, the heterogeneity of the study population, variety of the intensity, and duration of the tai chi interventions could affect the results [14].

Further, this review focuses on clinical studies investigating the effects of tai chi on metabolic diseases (Table 2).

Tsang *et al.* [16] conducted a single-blind, randomized controlled trial among 38 sedentary older patients with type 2 diabetes. Study participants completed supervised one-hour tai chi practices twice a week, for 16 weeks. The tai chi exercise consisted of 12 movements from the Yang and Sun styles. Static and dynamic balance abilities, maximal gait speeds, and habitual physical activity levels significantly increased in the tai chi group; however, there were no group effects found in the control group. Unfortunately, this study did not evaluate metabolic risk factors, such as blood glucose levels, lipid profiles, and blood pressure. The tai chi intervention for diabetes was not sufficient for the improvement of balance, physical performance, muscle performance, or HR-QOL in older patients with type 2 diabetes [17]. Zhang and Fu investigated the effects of the 24-style of tai chi on metabolic control in female patients with type 2 diabetes. Twenty subjects were randomly assigned to tai chi training and control groups. Subjects in the training group performed supervised tai chi exercises for 1 h/day, five days/week, for 14 weeks. After 14 weeks of tai chi exercises, fasting plasma glucose, glycated serum proteins, and triglycerides were reduced, whereas fasting plasma insulin levels increased. However, group effects between the tai chi training and control groups were not significant [18]. A recent study examined the long-term effects of tai chi on weight, blood pressure, lipid profiles, renal function, and quality of life. A total of 266 subjects with hypertension were randomly assigned to tai chi intervention and control groups. Subjects in the intervention group attended a tai chi exercise program for 12 months. Systolic blood pressure, diastolic blood pressure, and body mass index (BMI) significantly decreased after the tai chi intervention. Renal function, represented by estimated glomerular filtration rate (eGFR), was maintained, and HR-QOL improved; however, dyslipidemia and hyperglycemia did not improve. Additionally, there were significant between-group differences in terms of blood pressure, eGFR, and BMI. It appears that long-term tai chi exercise may be effective in the management of risk factors associated with hypertension. This is the first RCT to show the long-term effects of tai chi on metabolic diseases; however, details of the intervention, such as style, intensity, and duration of tai chi, were not described in the paper [19]. Liu *et al.* assessed the effects of tai chi on HR-QOL in individuals with impaired glucose tolerance and diabetes, using the Medical Outcomes Study 36-Item Short-Form Health Survey (SF-36), although metabolic parameters were not measured. Forty-one participants were randomly allocated to tai chi intervention and usual medical care control groups. Tai chi intervention was a supervised group program with 1.5 h of practice, three times/week, for 12 weeks. The exercise was KaiMai tai chi/qiong style. They noted that because of differences in individual health and fitness levels, the intensity of the exercise varied among the participants. There were significant between-group differences for the tai chi intervention group in physical functioning, role-physical, bodily pain, and vitality [6]. They previously reported that the tai chi exercise improved BMI, waist circumference, blood pressure, HbA1c, insulin resistance, stress, depressive symptoms, and the SF-36 score. However, this was a small, non-controlled study, and a single group before-after study [20]. Thus, it appears that tai chi improves physical QOL, but its beneficial effects on metabolic diseases are uncertain. Chen *et al.* conducted a RCT in a single center among 104 obese patients with type 2 diabetes. Fifty-six patients were allocated to the tai chi intervention group, and 48 patients were allocated to the conventional exercise group. The tai chi exercise session consisted of 30 min of Chen-style tai chi chuan 99-form, lasting up to 1 h, three times/week, for 12 weeks. Although no significant improvement in BMI, lipid profiles, or oxidative stress profiles was observed in the conventional exercise group, serum triglycerides, C-reactive protein, and malondialdehyde decreased, and high-density lipoprotein cholesterol increased in the tai chi intervention group. HbA1c values did not significantly decrease. The intensity of the exercise was monitored by the heart and respiration rates of the subjects during exercise, and both groups performed at a similar intensity (moderate intensity); however, there were between-group differences in changes of metabolic parameters. The authors stated that the key benefits of tai chi exercise do not depend on energy expenditure but on the efficacy of cardiopulmonary function, antioxidative action, and improved metabolism [8]. Although RCTs are the gold standard study design to reveal the efficacy of tai chi for metabolic diseases, there was considerable heterogeneity between the studies. Specifically, we should assess the methodological quality of each article to discuss the evidence of whether tai chi is really effective for improving metabolic diseases. Table 3 shows the methodological differences among the above-mentioned clinical studies.

There are interesting but non-randomized and non-controlled clinical studies that investigated the effects of tai chi on nerve function and immunity in patients with type 2 diabetes. Hung *et al.* evaluated changes in peripheral nerve modulation in 28 patients with type 2 diabetes and 32 healthy adult controls. All participants performed Cheng-style tai chi exercise for 40 min/session, three sessions/week, for 12 weeks. The relative intensity of the exercise was light to moderate, according to heart rate– and Borg rate–perceived exertion. After the 12-week tai chi exercise course, fasting blood glucose levels decreased in diabetic patients, as compared with healthy controls. No significant change in nerve conduction velocity, distal latency, or proximal and distal amplitude was found in the control group. However, the nerve conduction velocity of the tibial and median nerves, as well as the distal sensory latency of the ulnar nerves, in patients with type 2 diabetes improved after the 12-week tai chi exercise program [7]. This study suggests that tai chi may improve diabetic neuropathy as a result of microangiopathy. Nitric oxide may play an important role in the pathogeneisis of diabetic neuropathy [21] as shown in previous studies, where regular tai chi exercise was associated with the dilation of microvasculature mediated by nitric oxide [22,23]. Yeh *et al.* investigated the association between tai chi exercise and immunity in patients with type 2 diabetes. A before-after study among 32 patients with type 2 diabetes showed that the percentages of CD4 and CD8 lymphocyte subpopulations decreased, both proportional and absolute counts of CD4CD25 regulatory T-lymphocytes increased, and the proportional count of CD8CD28 cytotoxic lymphocytes decreased, along with the decrement of HbA1c levels, after a 12-week tai chi exercise regimen. Subjects performed Cheng-style tai chi 37-form, supervised by an expert, for 40 min/practice, three days/week, for 12 weeks. The study demonstrated that tai chi exercise could improve glucose metabolism, leading to a beneficial effect on immune regulatory functions in type 2 diabetic patients [24]. They also investigated the effect of a 12-week tai chi exercise regimen on helper T cell reactions in patients with type 2 diabetes. Thirty pairs of type 2 diabetic patients and age-matched controls participated in the study. After 12 weeks of tai chi exercise, the interleukin (IL)-12 levels increased and IL-4 levels decreased in the diabetic patients, but not in the controls. One of the Th1 transcription factors, T-bet m-RNA levels, also increased in type 2 diabetic patients. From this we may conclude that tai chi exercise may improve immunity as well as glucose metabolism [9].

## 4. Other Martial Arts

To the best of my knowledge, there are no systematic reviews or meta-analysis concerning the effects of other types of martial arts, such as judo, karate, tae kwon do, kung fu, or kick boxing, on metabolic diseases. A narrative review assessed the effect of such hard/external martial arts on physical fitness, including balance, flexibility, strength, cardiorespiratory fitness, and mental functions in people >40 years of age, but it failed to show sufficient evidence of the effect of martial arts on health because of the methodological shortcomings of previous clinical studies [25]. However, several clinical studies have shown significant associations between martial arts and metabolic diseases.

Tsang and her colleagues investigated whether kung fu, a Chinese martial art, was effective for improving body composition in overweight/obese adolescents, with a randomized placebo-controlled trial. A total of 20 subjects, aged six to 12 years, overweight/obese and sedentary, were investigated. Subjects were randomly assigned to kung fu or tai chi (control) groups, and they practiced each exercise three times, in 1 h sessions each week, for over six months. The kung fu training was based on the Choy Lee Fut Hung Sing Gwoon style. The resulting total body fat and abdominal fat mass, measured using dual-energy X-ray absorptiometry, physical fitness, muscle strength, and metabolic parameters, including HbA1c and serum lipid profiles, were assessed. Due to natural growth, their height, weight, bone mineral density, and lean mass increased during the study. However, total mass and abdominal fat mass were significantly reduced in both groups without a significant group effect. This study suggests that not only tai chi but also kung fu may be effective for the improvement of obesity in adolescents [26]. Kung fu training also resulted in greater improvements in submaximal cardiovascular fitness, lower muscle endurance, and upper body muscle velocity compared with those observed by tai chi training. The kung fu exercise improved physical fitness in overweight/obese adolescents with low physical fitness [27]. Moreover, although there were no between-group differences, HbA1c tended to decrease over time. Significant associations of increased lean body mass, with reductions in HbA1c, insulin resistance, triglycerides, and total cholesterol, suggest that the increase of lean mass by kung fu training may have favorable effects on metabolic health, independent of changes in fat mass [28]. Benbenek-Klupa *et al.* examined glycemic control in patients with type 1 diabetes who were engaged in combat sports. Three out of the five study participants were mixed martial arts competitors, and two were kick boxers. Each patient received multiple daily insulin injection therapy or continuous subcutaneous insulin infusion therapy, and glycemic control without hypoglycemia and hyperglycemia, pre- and post-combat sports, was expected to be very difficult. A detailed protocol for glycemic management was made in case of the occurrence of hypoglycemia and hyperglycemia related to performing combat sports, during the study period. In comparison with physically inactive control subjects, whose age, sex, and initial HbA1c were matched, the self-monitoring of blood glucose was more frequent. Glycemic control in all patients was improved or maintained, without diabetic ketoacidosis and severe hypoglycemia, during the follow-up. Type 1 diabetic patients who regularly engage in hard-contact martial arts can achieve or maintain satisfactory glycemic control without the increased risk of severe hyperglycemia and hypoglycemia if they have the access to expertise in physical activity and diabetes management. However, this is only a very small retrospective study, and a further prospective study will be needed for confirmation [29]. Benedini *et al.* reported metabolic and hormonal responses to a session of karate. Ten subjects practicing karate, with a mean training period of 10–12 h/week, were recruited. All subjects participated in two experimental exercises (3 min for kata and kumite) in a randomized crossover design. The kata session is a simulation of combat, combinations of punches and kicks, and a sequence of a pattern of fighting. The kumite session is fighting with punches and kicks against an opponent. Plasma glucose levels increased after the karate exercise, and after the kumite session, plasma glucose levels were significantly higher than those after the kata session. Epinephrine and norepinephrine also increased after the karate exercise. There were no significant changes in plasma insulin or cortisol levels; however, testosterone increased after the karate exercise. These results should not be applied to patients with metabolic diseases because the study subjects were healthy, elite karate athletes. The kumite exercise is not a favorable exercise for patients with diabetes, particularly with progressed diabetic complications, because an acute glycemic response to kumite could cause severe hyperglycemia in patients with diabetes [30]. A cross-sectional study investigated the association between martial arts and arterial stiffness. Douris *et al.* recruited 20 middle-aged subjects who were divided into martial arts and sedentary control groups. The martial artists practiced Soo Bahk Do, a traditional Korean martial art similar to karate, for approximately 20 years. The paper described that a typical Soo Bahk Do class lasted 1 h, but the intensity and frequency of the exercises were not described in detail. The martial artists had lower pulse wave velocity than did the sedentary control group. Trunks and hamstrings in the martial artists were also more flexible than in the controls, suggesting that martial arts training, with an emphasis on flexibility, is associated with less arterial stiffness [31]. Douris and his colleagues previously showed that Soo Bahk Do practitioners had higher levels of glutathione than the age-matched sedentary controls. This may demonstrate that martial arts could have a protective effect against oxidative stress [32]. Table 4 shows a summary of the above-mentioned clinical studies.

## 5. Adverse Effects of Martial Arts

The incidence of injuries and accidents associated with martial arts should also be discussed. Most of the tai chi styles are soft/internal styles, and the incidence rate of injuries tends to be small. Although tai chi seems to have no serious adverse effects, a systematic review have shown that it is associated with minor musculoskeletal pain (e.g., knee and back pain) [33]. Martial arts practiced as a full-contact style seems to have a high incidence rate of injuries. Several systematic reviews have shown that an average injury risk is approximately 11%–12% in judo [34], 2%–14% in taekwondo [35], and 23% in mixed martial arts [36] in young elite athletes. However, when martial arts are performed in a light or no-contact style, the incidence rate of injuries is low [37]. Zetaruk *et al.* conducted a retrospective cohort study, involving 263 martial arts practitioners, to analyze and compare five styles of martial arts with respect to injury incidents. The incidence rate of injuries was 59% for tae kwon do, 51% for aikido, 38% for kung fu, 30% for karate, and 14% for tai chi. No sex differences were found. Subjects >18 years of age (odds ratio = 3.95; 95% CI, 1.48–8.52) had >three years of experience (odds ratio = 2.46; 95% CI, 1.51–4.02), practiced for >3 h/week (odds ratio = 1.85; 95% CI, 1.13–3.05), and were associated with an increased risk of injury. Although the types of injuries varied depending on the styles of martial arts, martial arts seem to be safe for young individuals and beginners [38]. Considering that patients with metabolic diseases are relatively old, fragile, and inactive, the practice of a hard-contact style of martial arts should be avoided. However, no-contact and/or solo-practice martial arts, such as tai chi and kata of karate, can be performed without serious injuries. Such soft/internal styles of martial arts may be recommended as exercise for patients with metabolic diseases. Moreover, vigorous exercise may increase the risk of acute coronary events and sudden cardiac arrest in susceptible individuals who do not engage in regular exercise [39,40], and we should be careful when older patients with metabolic diseases perform martial arts. Umeda *et al.* [41,42] reported that energy restriction combined with intense exercise might have impaired muscle and immune function in judo athletes. We should also note whether individuals have adequate energy intake in martial arts participation. Combat sports usually have weight categories, and most elite athletes take drastic measures (e.g., starvation, restriction of fluid intake, and sweating) to achieve target body weight [43]. Indeed, the prevalence of rapid weight loss (typically 2%–10% weight reduction) in combat sports is as high as 50% [44], and it must be harmful for athletes’ health.

## 6. Conclusions

Systematic reviews of RCTs investigating the effectiveness of tai chi for metabolic diseases, including hypertension, dyslipidemia, and type 2 diabetes, showed that no sufficient evidence exists. However, when non-controlled studies, observational studies, and case series were included in this analysis, tai chi was found to be effective for reducing the risk of stroke and improving metabolic diseases. Other styles of martial arts, such as kung fu and karate, may be beneficial for body composition, glycemic control, and arterial stiffness; however, evidence is currently limited. The strength of this review is first to summarize the current literature in the effects of various styles of martial arts, not limited to tai chi, on metabolic diseases. Most previous studies evaluating the effects of martial arts on health were limited by a small numbers of subjects and short study durations. Previous RCTs also have methodological heterogeneity which makes it difficult to evaluate the efficacy of martial arts on metabolic diseases. Some confounding factors such as dietary intake may also affect the study results. For standardized evaluation of the quality and validity of clinical studies, clinicians should note how studies are conducted, including inclusion/exclusion criteria, sample size, randomization, and details of intervention and control group. Well-designed prospective studies, preferably RCTs with larger numbers of subjects, are warranted to clarify the effectiveness of martial arts for treating metabolic diseases. Another problem with past studies is a biased distribution of the ages of the subjects. There were few intervention studies investigating the effects of tai chi in children/adolescents or hard/external style martial arts in the elderly. Elderly patients with metabolic diseases should not perform a hard-contact style of martial arts against a real opponent because the incidence rate of injuries and accidents would be very high. However, they can practice a solo style of hard/external martial arts, under the supervision of experts in physical activity and metabolic diseases. In addition, the influence of a soft/internal style of martial arts, such as tai chi, on the growth of children provides an interesting perspective. Finally, traditional martial arts include unique forms of training, such as breathing techniques and manipulating bones and muscles. Investigating the clinical effects of such training on health, beyond the intensity and frequency of the exercise, may constitute a promising future study.

## Figures and Tables

**Table 1:** Excerpt of various sports activities with moderate intensity.

Sports	METs
Gymnastics, general	3.8
Golf, general	4.8
Tennis, doubles	4.5
Volleyball, non-competitive, general	3.0
Softball, practice	4.0
Bowling	3.8
Basketball, shooting practice	4.5
Martial arts, practice, slow pace	5.3

Each activity is quoted from Ainsworth *et al.* 2011 Compendium Physical Activities [2]. METs = metabolic equivalents.

**Table 2:** Clinical trials investigating the effects of tai chi on health in patients with metabolic diseases.

Authors, Year	Study Design	Subjects	Tai Chi Exercise (Style, Frequency, and Duration)	Results
Tsang *et al.*, 2007 [16]	RCT	38 patients with type 2 diabetes, ≥50 years of age	12 movements from Yang and Sun styles 1 h, 2 sessions/week, 16 weeks	No improvements in balance, physical performance, muscle performance and HR-QOL
Zhang and Fu, 2008 [18]	RCT	20 women with type 2 diabetes	24-style 1 h, 5 days/week, 14 weeks	Fasting plasma glucose↓, glycated serum proteins↓, triglycerides↓, and fasting plasma insulin↑. No significant group effects
Sun and Buys., 2015 [19]	RCT	266 subjects with hypertension	No descriptions of style 3 h/week in group and 2h/week at home, 12 months	Blood pressure↓, BMI↓, eGFR→, HR-QOL↑
Liu *et al.*, 2013 [6]	RCT	41 subjects with elevated blood glucose or diabetes	KaiMai tai chi/qiong style 1.5 h, 3 times/week,12 weeks	Physical QOL↑
Chen *et al.*, 2010 [8]	RCT	104 obese patients with type 2 diabetes	Chen style, 99-form 1 h, 3 times/week, 12 weeks	BMI↓, triglycerides↓, HDL-cholesterol↑ C-reactive proteins↓, malondialdehyde↓
Hung *et al.*, 2009 [7]	Before-after study	28 patients with type 2 diabetes and 32 healthy adult controls	Cheng style 40 min, 3 sessions/week, 12 weeks	Improvement of nerve conduction velocities in patients with type 2 diabetes
Yeh *et al.*, 2007, 2009 [9,24]	Before-after study	32 patients with type 2 diabetes 30 pairs of type 2 diabetic patients and controls	Cheng style, 37-form 40 min, 3 days/week, 12 weeks	Improvement of immunity

RCT = randomized controlled trial; HR-QOL = health related quality of life; BMI = body mass index; eGFR = estimated glomerular filtration rate; HDL = high density lipoprotein.

**Table 3:** Comparison of methodologies in RCTs investigating the effects of tai chi on health in patients with metabolic diseases.

Authors, Year	Inclusion Criteria	Exclusion Criteria	Subjects	Controls	Statistical Method
Tsang *et al.*, 2007 [16]	Type 2 diabetes ≥50 years of age ≤2 exercise sessions/week	Cognitive impairment (MMSE ≤ 24) Changes in medication Hip or knee arthritis Current tai chi participation Residence in a nursing home Amputation of a limb Severe visual impairment	Age: 65 ± 8 years Sex: 8 men, 30 women Ethnicity: 89.5% of subjects were Caucasian BMI: 32.2 ± 6.3 kg/m^2^ Dropout: One subject	Sham exercise (calisthenics and gentle stretching)	Described in detail
Zhang and Fu, 2008 [18]	Type 2 diabetes	Insulin therapy Exercise ≥2 times/week Resistance training participation Changes in medication Serum creatinine levels ≥200 M Proteinuria >1 g/day Blood pressure >160/95 mmHg	Age: 57.4 ± 6.2 years Sex: 20 women Ethnicity: Asian BMI: Not described Dropout: None	Free activity program	Not described in detail (e.g., How to randomize and calculate sample size)
Sun and Buys., 2015 [19]	Hypertension ≥45 years of age Residence in Changshu city	Not described in detail	Age: 66.5% of subjects aged 45–64 years, 33.5% of subjects aged over 65 years Sex: 48 men, 218 women Ethnicity: Asian BMI: 23.4 ± 3.1 kg/m^2^ Dropout: 30 subjects, 34 subjects were also excluded from analysis	Non-exercise-related activities such as reading and computing	Described in detail
Liu *et al.*, 2013 [6]	Type 2 diabetes or IFG and IGT No medication	Type 1 diabetes Diabetic medication BMI >45 kg/m^2^ Aged <30 years, >71 years Serious injury No time Residence not in Brisbane Blood glucose level unknown	Age: 59 ± 8 years Sex: 16 men, 25 women Ethnicity: Not described BMI: Not described Dropout: One subject	Usual medical care	Described in detail
Chen *et al.*, 2010 [8]	Type 2 diabetes (HbA1c > 7%) ≥40 years of age BMI ≥ 30–35	Serious operation Myocardial infarction Stroke Severe liver or kidney disease Gait disturbance Regular exercise ≥ 3 times/week	Age: 59.1 ± 6.2 years (tai chi group), 57.4 ± 5.8 years (control group) Sex: 45 men, 59 women Ethnicity: Asian BMI: 33.5 ± 4.7 kg/m^2^ (tai chi group), 33.2 ± 4.1 kg/m^2^ (control group) Dropout: 13 subjects	Conventional exercise (aerobic dance)	Described in detail

RCT = randomized controlled trial; MMSE = mini mental state examination; BMI = body mass index; IFG = impaired fasting glucose; IGT = impaired glucose tolerance.

**Table 4:** Clinical studies investigating the associations of martial arts with metabolic parameters.

Authors, Year	Study Design	Subjects	Martial Arts (Style, Frequency and Duration)	Results
Tsang *et al.*, 2009 , 2010 [26,27]	Randomized placebo-controlled trial	20 overweight/obese subjects, aged 6 to 12 years	Kung fu 1 h, 3 times/week, over 6 months	Total and abdominal fat mass↓(without group effect) Submaximal cardiovascular fitness↑, lower muscle endurance↑, upper body muscle velocity↑ Lean body mass↑, HbA1c↓, insulin resistance↓, triglycerides↓, total cholesterol↓
Benbenek-Klupa *et al.*, 2015 [29]	Case-control study	5 patients with type 1 diabetes, aged 18 to 34 years	Mixed martial arts and kick boxing	Improvement or maintenance of glycemic control without diabetic ketoacidosis and severe hypoglycemia during at least a 2-year follow-up
Benedini *et al.*, 2012 [30]	Randomized crossover study	10 healthy subjects, university students	Karate (*kata* and *kumite* sessions)	Plasma glucose levels↑ Epinephrine↑, norepinephrine↑, testosterone↑
Douris *et al.*, 2009 [32]	Cross-sectional study	10 martial artists and 10 sedentary subjects, middle-aged	Soo Bahk Do	Lower pulse wave velocity in martial artists
Douris *et al.*, 2013 [31]	Matched-pair design	9 martial artists and 9 sedentary subjects, middle-aged	Soo Bahk Do	Higher levels of glutathione and lower levels of glutathione disulfide in martial artists

HbA1c = hemoglobin A1c.

## Abbreviations

The following abbreviations are used in this manuscript:

METs	metabolic equivalents
HR-QOL	health-related quality of life
CVD	cardiovascular disease
RCT	randomized controlled trial
CI	confidence intervals
eGFR	estimated glomerular filtration rate
